# Human sex hormone-binding globulin gene expression- multiple promoters and complex alternative splicing

**DOI:** 10.1186/1471-2199-10-37

**Published:** 2009-05-05

**Authors:** Atif M Nakhla, Daniel J Hryb, William Rosner, Nicholas A Romas, Zhaoying Xiang, Scott M Kahn

**Affiliations:** 1 Department of Urology, Columbia University, New York, NY, 10032, USA; 2 Institute for Health Sciences, St Luke's-Roosevelt Hospital, 432 W 58th St Room 405, New York, NY, 10019, USA; 3 Department of Medicine, Columbia University, New York, NY 10032, USA; 4 Herbert Irving Comprehensive Cancer Center, Columbia University, New York, NY 10032, USA; 5 Department of Microbiology and Immunology, Weill Cornell Medical College, New York, NY 10021, USA

## Abstract

**Background:**

Human sex hormone-binding globulin (SHBG) regulates free sex steroid concentrations in plasma and modulates rapid, membrane based steroid signaling. SHBG is encoded by an eight exon-long transcript whose expression is regulated by a downstream promoter (P_L_). The SHBG gene was previously shown to express a second major transcript of unknown function, derived from an upstream promoter (P_T_), and two minor transcripts.

**Results:**

We report that transcriptional expression of the human SHBG gene is far more complex than previously described. P_L _and P_T _direct the expression of at least six independent transcripts each, resulting from alternative splicing of exons 4, 5, 6, and/or 7. We mapped two transcriptional start sites downstream of P_L _and P_T_, and present evidence for a third SHBG gene promoter (P_N_) within the neighboring FXR2 gene; P_N _regulates the expression of at least seven independent SHBG gene transcripts, each possessing a novel, 164-nt first exon (1N). Transcriptional expression patterns were generated for human prostate, breast, testis, liver, and brain, and the LNCaP, MCF-7, and HepG2 cell lines. Each expresses the SHBG transcript, albeit in varying abundance. Alternative splicing was more pronounced in the cancer cell lines. P_L_- P_T_- and P_N_-derived transcripts were most abundant in liver, testis, and prostate, respectively. Initial findings reveal the existence of a smaller immunoreactive SHBG species in LNCaP, MCF-7, and HepG2 cells.

**Conclusion:**

These results extend our understanding of human SHBG gene transcription, and raise new and important questions regarding the role of novel alternatively spliced transcripts, their function in hormonally responsive tissues including the breast and prostate, and the role that aberrant SHBG gene expression may play in cancer.

## Background

Sex Hormone-Binding Globulin (SHBG) is a multifunctional protein that influences androgen and estrogen action in humans on at least two levels. In plasma, SHBG is the major sex steroid-binding protein, regulating their availability to responsive tissues. SHBG is also an integral part of a membrane-based steroid signaling pathway in certain responsive tissues, including the prostate and breast.

The initial steps of SHBG-mediated steroid signaling in the prostate and breast have been well characterized in cultured cells and tissue explants [1,2]. Unliganded SHBG binds to a specific membrane receptor (R_SHBG_); the SHBG-R_SHBG _complex is activated by subsequent binding of an appropriate androgen or estrogen, independently of the androgen or estrogen receptors [3]. R_SHBG _activation leads to a rapid increase in intracellular cAMP [4,5], presumably through the action of a G protein [6]. Studies in cultured cells have demonstrated downstream effects that include protein kinase A activation [7], increased prostate specific antigen expression [8], decreased progesterone receptor expression [9], induced apoptosis [10], and seemingly disparate findings of reduced MCF-7 breast cancer cell growth [11-13] and increased ALVA-41 prostate cancer cell growth [14]. However, a biologic role of R_SHBG _signaling in the intact prostate and breast awaits demonstration.

The source of the SHBG that initializes R_SHBG _signaling *in vivo *is unclear; it could be taken up from the plasma, where it is relatively abundant, or synthesized in cells in which signaling occurs. In addition to the liver which is well known to be the source of plasma SHBG, and testis (which synthesizes the differentially glycosylated SHBG isoform, androgen binding protein (ABP), SHBG protein and mRNA have been demonstrated in other human tissues, including the prostate and breast [15-19]. The relatively stable concentration of plasma SHBG [20] makes it a less likely source for the initiation of R_SHBG _signaling, as the physiology of important signaling molecules depends on their variation with time. Other, more variable sources of SHBG would be better candidates. This raises the possibility that the prostate and breast themselves could control local SHBG concentrations, thereby regulating R_SHBG _activation in an autocrine/paracrine manner.

**Figure 1:**
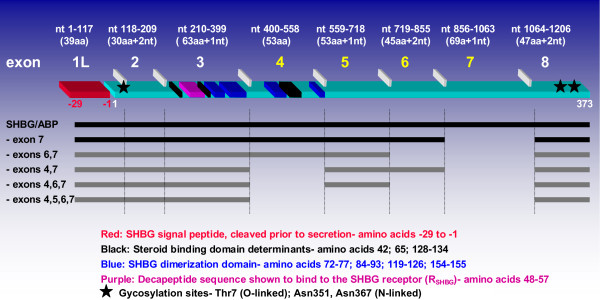
**Schematic diagram of the contiguous human SHBG/ABP transcript and alternatively spliced transcripts arising from the downstream promoter, P_L_**. A structure/function diagram for the eight exon long SHBG/ABP transcript is shown above. The nucleotide positions of each exon are at the top (the exon 1 start codon defined as beginning with nucleotide +1); below that, the number of amino acids encoded by each exon. Conserved exons are numbered in white, alternatively spliced exons in yellow. Exon boundaries are denoted by horizontal white rectangles. Shown in red is the region of the SHBG/ABP transcript encoding the 29 amino acid long signal peptide, in light blue, the 383 amino acid long SHBG/ABP monomer. Steroid binding determinants in the mature SHBG protein are denoted by black rectangles, those forming the dimerization domain are blue, and that encoding the decapeptide sequence known to mediate binding of SHBG to R_SHBG _is purple. Below, in black, are linear depictions of the two previously described human SHBG gene transcripts originating from P_L_; the four novel transcripts described in this study are depicted in grey. Transcripts are identified on the left by missing exons. Three of five alternatively spliced P_L _transcripts retain the SHBG/ABP reading frame, whereas those lacking exon 7 alone and exons 4–7 undergo identical frameshifts within exon 8 that may affect their inherent stabilities (see discussion).

Most work regarding human SHBG mRNA expression has been based on earlier reports of the existence of two major and two minor transcripts [21-26] (GenBank Accession Number: M31651). A downstream promoter, (P_L_), regulates the expression of a major, eight-exon mRNA transcript that encodes SHBG (NM_001040)(Figure 1). The liver secretes SHBG [27]; the nascent SHBG translation product is a 402 amino acid precursor protein which is glycosylated and cleaved at its amino terminus to remove a 29 amino acid long leader peptide [21,22,28] leading to the secretion of mature SHBG into plasma. Domains responsible for steroid binding and dimerization are encoded within exons 3 and 4[29,30], while binding of SHBG to R_SHBG _is mediated by a decapeptide sequence encoded within exon 3 [31]. Mature SHBG has an O-linked glycosylation site at Thr7, and N-linked sites at Asn 351 and Asn 367 [32,33]. The identical eight exon transcript is also expressed in the testis, which is translated into ABP [23,25]. In addition to the SHBG/ABP transcript, there is a second major SHBG gene transcript in the testis (X16350, X16351) derived from a second promoter (P_T_), located 1.9 kb upstream of P_L _[22]. It possesses unique 5' end sequences, referred to here as exon 1T, and lacks exon 7. Transgenic mice express a human SHBG species that appears to be attributable to this transcript [34], however, such an isoform has not been identified in humans. In addition to the two major transcripts, two minor human SHBG gene transcripts resulting from alternative splicing of exon 7 have been reported [35]. These include an SHBG/ABP-like transcript that lacks exon 7 (Figure 1), and an eight exon long transcript originating from P_T _that retains exon 7.

The LNCaP (prostate cancer) and MCF-7 (breast cancer) cell lines express SHBG at the mRNA and protein levels and possess R_SHBG _activity[12,17-19], making them attractive *in vitro *models for studying the effects of local SHBG expression on steroid signaling and R_SHBG _regulation. These cell lines retain both alleles of the p53 gene [36], located ~35 kb from the distal end of the SHBG gene locus on chromosome 17p13.1 and, due to this extremely close linkage, we presumed that both SHBG alleles also remain intact. We initially intended to confirm prior reports of SHBG mRNA expression in these cell lines as a prelude to investigating the function of locally expressed SHBG in the prostate and breast. As positive controls for SHBG mRNA expression, we included the human HepG2 (hepatoma) cell line (known to express abundant amounts of the SHBG/ABP transcript) and normal testis tissue (known to express abundant amounts of the major P_T_-derived transcript).

In the course of that effort, we found that transcription of the human SHBG gene is far more complex than had been previously reported. We performed a detailed structural analysis of human SHBG transcripts from the human LNCaP, MCF-7, and HepG2 cell lines, and from normal human liver, testis, prostate, breast, and brain tissues. RT-PCR based mapping assays revealed the existence of at least three distinct human SHBG gene promoters; we determined transcriptional start sites for each. In addition to the four previously reported transcripts, we identified and characterized 15 novel SHBG gene transcripts resulting from alternate splicing of exons 4, 5, 6, and/or 7. Real-time, quantitative PCR showed that SHBG transcription is tissue dependent, and perhaps, cancer dependent. We also detected a smaller sized immunoreactive SHBG species in LNCaP, MCF-7 and HepG2 cells, which raises the possibility that at least one of these transcripts is translated. Based on our current knowledge of SHBG function, these results could have important biologic implications for the modulation of R_SHBG _activity and cellular steroid signaling.

## Results

### SHBG exon 5–8 RT-PCR

Because the longest run of A residues within the human SHBG coding region is only three, an oligo-d(T) primer was selected for first strand cDNA synthesis from LNCaP, MCF-7 and HepG2 cells, and from normal testis. As a control for the initial SHBG RT-PCR assays, first strand cDNA was also synthesized from MCF-7-Tr, a stably transfected cell line that constitutively overexpresses relatively high amounts of the contiguous exon 1L-8 transcript containing the human SHBG/ABP coding region.

**Figure 2:**
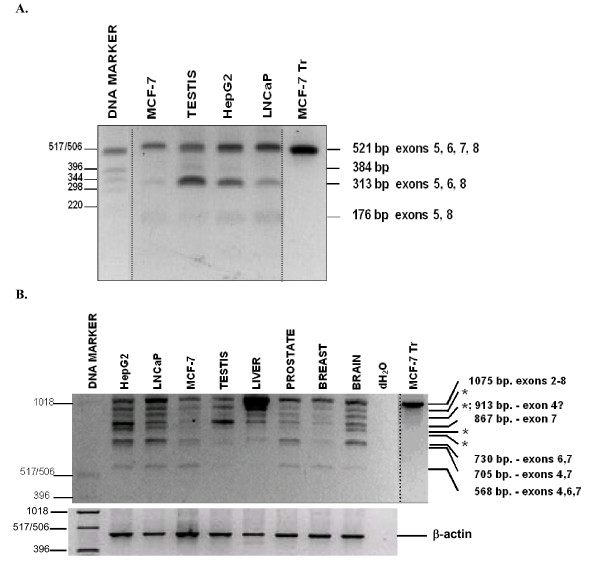
**Short range RT-PCR assays for human SHBG gene expression**. A. SHBG exon 5–8 assay. First strand cDNAs were generated from MCF-7, MCF-7-Tr LNCaP, HepG2 cells and normal human testis tissue using an oligo dT primer (see methods). Three RT-PCR transcripts were generated in the MCF-7 and LNCaP lanes, and four in the HepG2 and testis lanes. MCF-7-Tr is an MCF-7 clone stably transfected with a plasmid constitutively expressing large amounts of SHBG_L_. All bands were reamplified and sequenced. SHBG RT-PCR transcript structures are given on the right. Note- this is a merged figure- the DNA marker and transfection control lanes were consolidated from a different part of the gel. B. SHBG exon 2–8 assay. PCR primers, specific for exon 2 and exon 8, were used to amplify cDNAs prepared from HepG2, LNCaP, MCF-7, MCF-7-Tr, and normal human testis, liver, prostate, breast, and brain tissues. All bands from HepG2, LNCaP, MCF-7, and testis were reamplified and sequenced. Bands labeled "*" produced sequences that were inconsistent with the sizes of the transcripts, and which were identical to other characterized sequences. DNA marker sizes, in base pairs, are given on the left. RT-PCR fragment transcript structures and sizes are given on the right. Note- this is a merged figure- the transfection control lane was taken from a different part of the gel.

A short SHBG RT-PCR assay, spanning exons 5 through 8 [17], was used to generate partial SHBG mRNA expression profiles for LNCaP, MCF-7, HepG2, testis and the MCF-7-Tr control. Based upon prior reports of SHBG transcription (see Introduction), two RT-PCR fragments were anticipated from LNCaP and MCF-7 cells, a contiguous 521-bp fragment that retains exon 7, and a smaller 313-bp fragment that lacks exon 7. Instead, LNCaP and MCF-7 gave rise to three distinct RT-PCR fragments (Figure 2A). All 521-bp, 313-bp, and 176-bp RT-PCR bands were isolated, reamplified, and sequenced. In addition to the expected 521-bp and 313-bp RT-PCR fragments, whose identities were confirmed by sequencing, we also found a 176-bp RT-PCR fragment of low abundance. The 176-bp fragment was derived from a transcript(s) in which exon 5 is directly spliced to exon 8 (sequence analysis). This same 176-bp fragment was generated from HepG2 and normal testis. HepG2 and normal testis RT-PCR also revealed a faint, intermediate RT-PCR fragment of approximately 384-bp. We were unable to reamplify this fragment and its identity, therefore, is not established. As expected, the MCF-7-Tr RT-PCR control gave rise only to the contiguous 521-bp fragment, undoubtedly because the highly overexpressed full-length SHBG/ABP cDNA transcript overwhelmed the RT-PCR assay. The relative intensities of the 521-bp and 313-bp bands varied dependent on the source of the RNA. (Figure 2A).

### SHBG exon 2–8 RT-PCR

**Figure 3:**
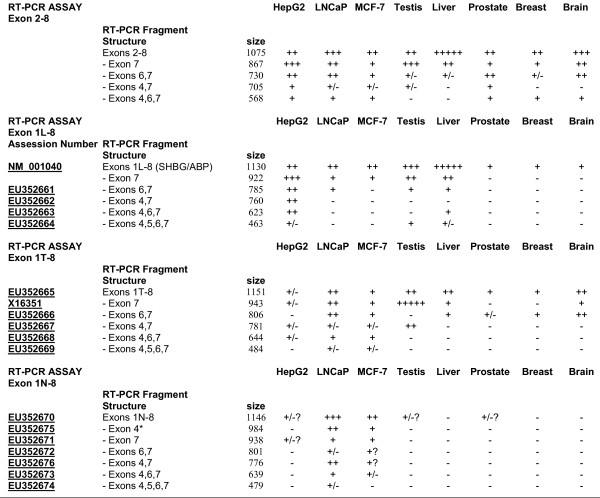
**Summary of RT-PCR results**. Semiquantitative representation of the relative amounts of each RT-PCR fragment. For ease of comparison, the abundance of each RT-PCR fragment in the exon 2–8 and the exon 1–8 RT-PCR assays has been assigned a value, denoted by the following- "+++++" most abundant, down to "+" detectable, "+/-" barely detectable, and "-" not detectable. For each individual assay, transcript structures were determined by sequence analysis of designated RT-PCR fragments (see Results). These were used to assign structures to similar sized RT-PCR fragments from remaining samples. *: This band was a mixture of 1N-8-exon 4 sequences and those of an ambiguous sequence. "?"-inconsistent appearance of fragment, presumably due to low abundance.

An exon 2–8 RT-PCR assay was designed to screen for alternative splicing of downstream exons, 3–7, in a single reaction, intentionally bypassing the assessment of individual contributions from P_L _and P_T_. First strand cDNAs, generated from normal liver, prostate, and breast, as well as from brain (which expresses SHBG [37-39], were also included as templates for RT-PCR. Adding further complexity to the picture of SHBG gene expression, the exon 2–8 RT-PCR assay generated nine distinct RT-PCR fragments (Figure 2B; summarized in Figure 3). Each HepG2, liver and testis RT-PCR fragment was reamplified and sequenced. The largest fragment, 1075-bp, contained the expected contiguous exon 2–8 sequence. Four of the remaining eight RT-PCR fragments were comprised of sequences that were consistent with their sizes. From largest to smallest, these transcripts lacked exon 7 (867-bp); exons 6 and 7 (730-bp); exons 4 and 7 (705-bp); and exons 4, 6 and 7 (568-bp). That exon 4 undergoes alternative splicing was confirmed using an RT-PCR assay that spanned exons 3 and 5 (data not shown). A prior report similarly found alternative splicing of exon 4, using an RT-PCR assay that included primers specific for exons 3 and 7 [40]. For reasons that are unclear, the four remaining RT-PCR fragments consistently gave sequences that were identical to those described above. For example, we had expected the ~913-bp RT-PCR fragment to give rise to a sequence derived from a transcript lacking exon 4, however, it gave the 1L-8 sequence lacking exon 7. As described below, given our positive identification of an exon 1N-8 transcript lacking exon 4, we suspect that technical factors (perhaps the generation of single stranded species during the PCR process) may have interfered with our ability to positively detect this specific transcript in the 2–8, 1L-8 and 1T-8 RT-PCR assays. As expected, only a single, intense contiguous exon 2–8 RT-PCR fragment was generated from MCF-7-Tr. The same individual exon 2–8 RT-PCR fragments were generated from all three cell lines, albeit in unequal abundances (Figure 2B).

### Identification of a novel SHBG promoter and transcriptional start sites by RACE analysis

Before assessing the splicing patterns of full-length SHBG transcripts, we used a nonbiased approach to determine whether additional human SHBG gene promoters exist along with P_L _and P_T_. In the rat, an alternative upstream SHBG promoter, P_A_, appears to share promoter elements with the rat FXR2 gene promoter [41]; it was speculated to have an active human homologue [42].

**Figure 4:**
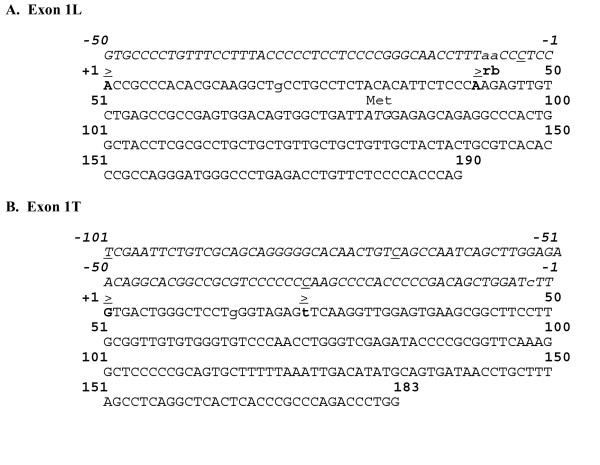
**Configuration of A) human SHBG exon 1L and B) exon 1T**. Transcriptional start sites, as determined in this study, are in bold letters and are denoted by a "≥ ". For both exons, position +1 is assigned to the distal, upstream start site. The exon 1L proximal start site is at position +42, the exon 1T proximal start site is at position +23. Upstream sequences are in italics. Previously reported human transcriptional start sites are underlined; not shown are two additional exon 1L start sites mapped at positions -114 and -125 [22]. Transcriptional start sites mapped in transgenic mice are in lower case letters [23]. "r" and "b" denote the corresponding positions of the major exon 1L rat [43] and rabbit [44] transcriptional start sites, respectively.

Transcriptional start sites downstream of SHBG gene promoters were identified using RLM-RACE ("RNA Ligase Mediated-Rapid Amplification of cDNA Ends"), a modified RACE assay which included steps specifically designed to prime only full length transcripts that possess 5' end cap structures. Beginning with total cellular RNAs isolated from LNCaP, HepG2, and normal testis, multiple RLM-RACE bands were generated from each sample (data not shown), and each band was reamplified and sequenced. The largest HepG2 band was actually a mixture of two closely sized RACE fragments. Distinct exon 1L 5' end boundaries mapped to A residues respectively located 79- (EU352659) and 38-nt (EU352660) upstream of the ATG start codon, indicating that P_L _utilizes at least two transcriptional initiation sites (Figure 4A). The proximal initiation site is consistent with positions of the major exon 1L rat and rabbit SHBG transcriptional initiation sites [41,43,44]. Our results differ from those human exon 1L transcriptional start sites originally determined [22], and with those mapped in the liver and kidney of mice expressing a human SHBG transgene [45].

The largest RLM-RACE band from normal testis was also a mixture of two similarly sized fragments, both of which contained exon 1T sequences (Figure 4B). The longer sequence extended to a G residue located 183-nt upstream of the exon 1T splice donor site (EU352657), whereas the shorter fragment extended to a T residue located 161-nt upstream (EU352658) of the splice donor site. These results are consistent with primer extension studies in the testis of a mouse expressing a human SHBG transgene, where major exon 1T initiation sites were located 161, 169, and 186-nt upstream of the exon 1T splice donor site [45]. Our results confirm that exon 1T lacks a potential ATG start codon.

**Figure 5:**
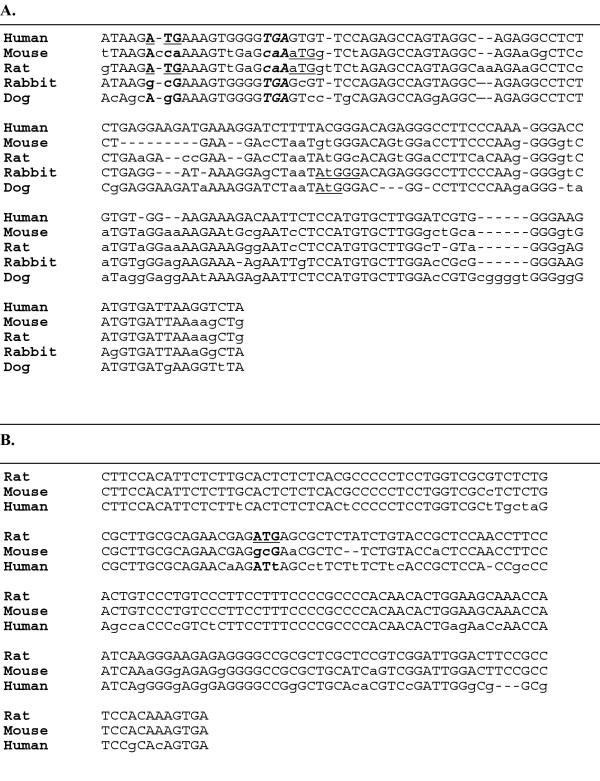
**A- Sequence of human SHBG exon 1N and species alignment**. Shown is an alignment between the full human SHBG exon 1N sequence and homologous sequences from mouse, rat, rabbit and dog obtained from Genbank. The putative human ATG start codon is in bold and underlined. The TGA stop codon is in bold and italics. Conserved nucleotides between species are capitalized, differences are in lower case letters. The human exon 1N open reading frame is predicted to be only 3 amino acids long. B- Sequence of rat SHBG exon 1A and species alignment. Shown is an alignment between the rat SHBG exon 1A sequence and homologous human and mouse sequences obtained from Genbank. The putative rat ATG start codon is in bold and underlined. Conserved nucleotides between species are capitalized, differences are in lower case letters.

LNCaP gave rise to a RACE fragment consisting of a novel, 164-bp sequence (EU352656) spliced directly to SHBG exon 2. This sequence maps to an uninterrupted genomic locus located 17 kb upstream of P_L _on chromosome 17p13.1, within the first intron of the fragile X mental retardation-related 2 (FXR2, NM_004860) gene, and is referred to here as SHBG exon 1N (Figure 5A). Exon 1N is distinct from the human counterpart of rat exon 1A (Figure 5B) (AF044263), located on the opposite side of the FXR2 gene promoter. We confirmed the 1N transcript initiation site in LNCaP cells in a second RACE assay, using a downstream primer complementary to 3' end sequences within exon 1N and the same 5' adaptor based primer (data not shown). A short exon 1N-2 RT-PCR assay was also performed on LNCaP, MCF-7, and HepG2 to confirm that exon 1N is incorporated into mature SHBG mRNA transcripts. A single RT-PCR fragment of the expected size was generated from LNCaP and MCF-7, but not from HepG2 (data not shown). All other RACE bands generated from LNCaP, HepG2 and normal testis were either comprised of SHBG sequences that had recombined with sequences from other chromosomes or were not specific for SHBG, and were considered to be experimental artifacts. This experimental approach does not rule out the possibility that additional human SHBG gene promoters may still exist.

### SHBG exon 1L-, 1T-, and 1N-8 RT-PCR

**Figure 6:**
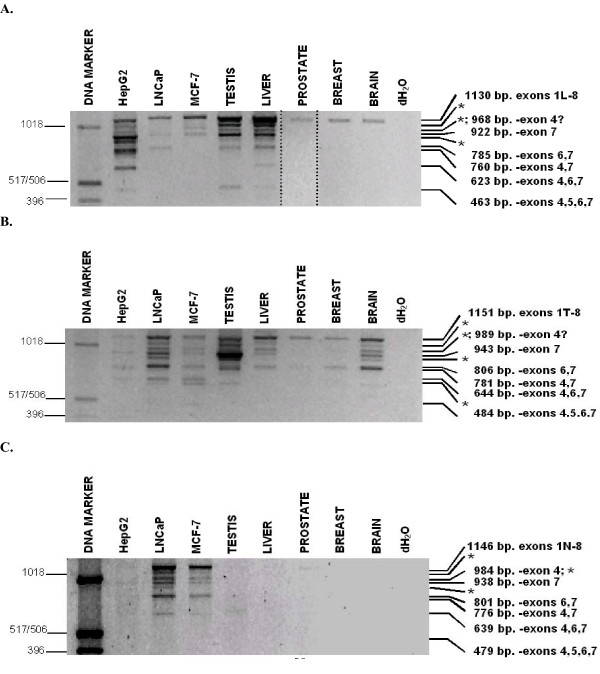
**RT-PCR analysis of transcripts including exon 1L (top), exon 1T (middle), and exon 1N (bottom)**. Separate individual RT-PCR amplifications were performed using 5' primers specific for exon 1L, 1T, and 1N, and a common exon 8 3' primer. Products were electrophoresed through a 1% agarose gel. All fragments were reamplified and sequenced. Bands labeled "*" produced ambiguous sequences that were inconsistent with the sizes of the transcripts, and which were identical to other characterized sequences (the smallest "*" fragment in the 1T-8 assay gave an unrelated sequence). DNA marker sizes, in base pairs, are given on the left. RT-PCR fragment transcript structures and sizes are given on the right. Note- 5A is a merged figure from two experiments to more clearly show the prostate 1L-8 RT-PCR fragment. Certain HepG2, MCF-7, testis and prostate fragments showed varied appearance in the 1N-8 assay, presumably due to their low relative abundance (Figure 3).

A comprehensive structural analysis of human SHBG gene transcripts was performed using exon 1L-8, 1T-8, and 1N-8 RT-PCR assays. These incorporated upstream primers specific for either exons 1L, 1T, or 1N and a common, downstream exon 8 primer. For these RT-PCR experiments, because exon 8 is the last described exon in the human SHBG gene, and it contains appropriate transcriptional processing sequences, the use of a reverse primer complementary to exon 8 sequences demonstrates the likely presence of full length transcripts. For the most part, counterparts of all exon 2–8 assay RT-PCR fragments appeared in one or more of the exon 1–8 assays (Figure 6; summarized in Figure 3). Overall, normal liver, prostate, and breast expressed a lower proportion of alternatively spliced SHBG transcripts than did the HepG2, LNCaP, and MCF-7 cell lines.

Nine individual RT-PCR fragments were produced in the exon 1L-8 assay (Figure 6A). Those generated from HepG2 were sequenced, as were all the 1130 bp fragments. The sizes of six of the nine HepG2 fragments were consistent with their sequences, whereas three were not, and remain unattributable. Sequence analysis of all 1130-bp fragments confirmed that they were derived from the contiguous SHBG/ABP transcript. This fragment was the sole detectable 1L-8 species in prostate, breast and brain. HepG2 also displayed 1L-8 RT-PCR fragments corresponding to smaller sized, alternatively spliced transcripts (Figure 1) that lacked exon 7, exons 4 and 7 (EU352662), exons 6 and 7 (EU352661), exons 4, 6, and 7 (EU352663), and a novel, 463-bp RT-PCR fragment. The 463-bp fragment was comprised of exon 1L, 2, 3, and 8 sequences (EU352664), representing a transcript lacking exons 4–7. To our knowledge, this is the first account of a human SHBG transcript that lacks exon 5 sequences. Alternatively spliced 1L-8 species were also observed in liver, testis, MCF-7 and LNCaP (sequence analysis confirmed that the relatively abundant 922 bp fragment in testis and liver corresponded to the transcript lacking exon 7). The sequences obtained for the remaining three different sized RT-PCR fragments unexpectedly gave sequences that were identical to specific SHBG transcripts described above. Because of the incongruence of their sizes and sequences, and despite repeated attempts at their resolution, their significance remains uncertain.

The exon 1T-8 assay produced a total of ten individual RT-PCR fragments (Figure 6B). Those generated from LNCaP and testis were reamplified and sequenced, as were all of the 1152 bp fragments. Six of the ten fragments LNCaP and testis fragments gave sequences that corresponded with their sizes. As expected, the 943-bp fragment, corresponding to the major exon 1T-8 transcript lacking exon 7, was the most abundant testis transcript. This transcript was found in all samples other than for prostate and breast. The contiguous exon 1T-8 transcript (EU352665) was present in all samples tested. Alternatively spliced exon 1T-8 transcripts lacking exons 6 and 7 (EU352666), 4 and 7 (EU352667), and 4, 6, and 7 (EU352668) were also detected. Testis and liver generated a very weak 484-bp 1T-8 fragment that was found by sequence analysis to consist only of exons 1T, 2, 3, and 8 (EU352669). Of the four remaining 1T-8 RT-PCR fragments, the smallest was unrelated to any SHBG transcript, while the remaining three fragments gave previously characterized SHBG transcript sequences that were incongruent with their sizes. Sequence analysis confirmed that all 1152 bp fragments from the remaining samples corresponded to the contiguous 1T-8 transcript. Furthermore, the sequences of selected abundant bands from the 1T-8 assay (the 806 bp liver and brain, the 622 bp MCF-7, the 484 bp MCF-7), also confirmed their structures.

The exon 1N-8 assay produced a total of nine individual RT-PCR fragments (Figure 6C). RT-PCR fragments from LNCaP and MCF-7 were reamplified and sequenced. Six of the nine fragments gave sequences that corresponded with their sizes. The contiguous 1146-bp 1N-8 fragment (EU352670) was present in LNCaP and MCF-7, and was faint in testis and prostate. The third largest fragment was found to consist of a mixed sequence derived from two transcripts, an appropriately sized 984-bp fragment that had undergone splicing of exon 4 alone (EU352675) and an aberrantly migrating fragment that lacks only exon 7. This result suggests that transcripts lacking exon 4 may have gone undetected in the 1L-8 and 1T-8 assays if their sequence signals were drowned out by more abundant co-migrating species. The 1N-8 assay also generated appropriately sized fragments corresponding to transcripts that have undergone splicing of exon 7 (EU352671), and exons 6 and 7 (EU352672), 4 and 7 (EU352676), 4, 6, and 7 (EU352673), and exons 4, 5, 6, and 7 (EU352674) (the latter did not reproduce well in Figure 6C). The reproducibility of the testis and HepG2 assays was inconsistent, likely due to the low abundance of 1N-8 transcripts in these samples.

### Quantitative PCR (qPCR) analysis of SHBG transcript abundance in human tissues and cell lines (Figure 7)

**Figure 7:**
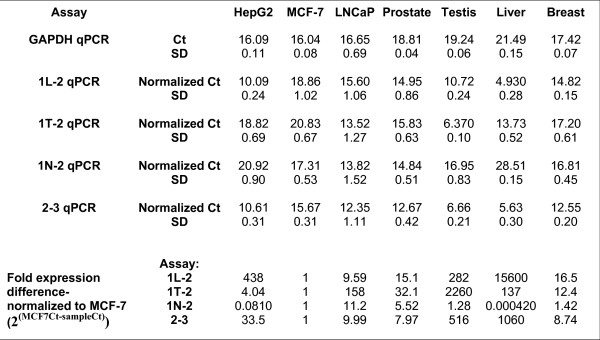
**Relative SHBG Gene Expression (Total and Promoter-specific) Among Human Cancer Cell Lines and Normal Tissues**. Quantitative RT-PCR (qPCR) was performed on total cellular RNA samples isolated from the indicated cell lines (in triplicate) and from normal tissues (in duplicate), using primers specific for SHBG exons 1L and 2, 1T and 2, 1N and 2, or exons 2 and 3 as described in the Experimental Procedures. Each sample was analyzed by qPCR in triplicate (nine total measurements for each cell line per assay, six for each tissue). Ct values represent the number of cycles required to reach an arbitrary point on the exponential part of the qPCR curves. "Normalized Ct" values for each of the 1L-2, 1T-2, 1N-2 and 2–3 assays represent mean Ct values for a given sample, normalized to the mean GAPDH Ct value for that same sample. SD: Standard deviation, calculated from the averages of the total measurements. Relative transcript abundance is presented as the ratio of Ct means; an arbitrary value of 1 is assigned to MCF-7.

Four independent real time, qPCR assays were performed to determine total relative SHBG mRNA abundance, and the relative abundance of transcripts derived from each of the three promoters. Total SHBG transcript abundance was measured by targeting common sequences within exons 2 and 3 that are present within all the SHBG transcripts identified in this study. Contributions from individual promoters were measured using qPCR assays specific for exons 1L, 1T, and 1N, respectively. The samples were normalized against GAPDH expression.

Overall, the exon 1L, and 1T, qPCR assays gave results that were comparable to the three individual exon 1–8 RT-PCR assays. Among the normal tissues, overall SHBG gene transcription, as measured by the exon 2–3 qPCR assay, was highest in liver and testis. Transcripts containing exon 1L were most abundant in liver, whereas those containing exon 1T were most abundant in testis. All normal tissues, other than liver, expressed transcripts containing exon 1N. Compared to their respective parental tissues, the three cancer cell lines all displayed reduced expression of exon 1L-containing transcripts, whereas there were varied differences in the abundance of exon 1T- and 1N-containing transcripts. Apparent discrepancies between certain exon 1N qPCR and RT-PCR results could signify that novel 3' end sequences may be incorporated into a fraction of SHBG gene transcripts; this would have gone undetected here due to our exclusive use of an exon 8-based downstream primer in longer range RT-PCR assays. Thus, actual human SHBG gene expression may be even more complex than is presented here.

### Initial Western blot analysis of SHBG protein expression in HepG2, LNCaP, and MCF-7 cells

**Figure 8:**
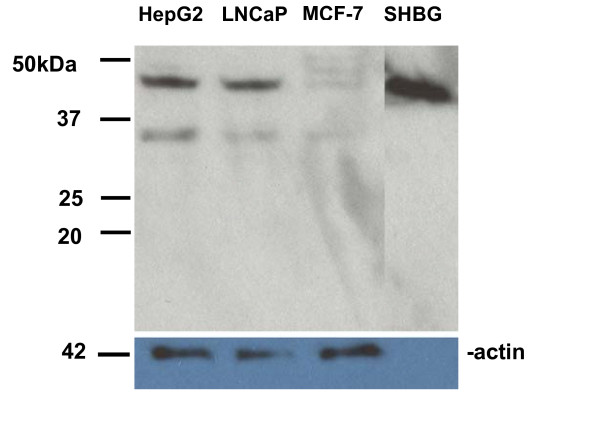
**Western blot analysis of SHBG protein expression in HepG2, LNCaP, and MCF-7 cells**. Protein extracts (20 μg each) prepared from HepG2, LNCaP, and MCF-7 cells, and 10 picograms of purified SHBG protein isolated from human plasma using a steroid affinity column, were electrophoresed through 10% LongLife polyacrylamide gels (Gradipore-VWR), transferred to PVDF membranes, and hybridized to either a rabbit anti-human SHBG polyclonal antibody (top, WAK-S1012-53, WAK-Chemie, Germany) or a rabbit anti-actin affinity purified polyclonal antibody (bottom, A2066, Sigma). Molecular weight (in kilodaltons) marker positions are given on the left. The top band in HepG2 and LNCaP cells migrates with a molecular weight of approximately 44–46 kD, while the bottom band migrates with a molecular weight of approximately 33–35 kD. The two larger bands in MCF-7 migrate with molecular weights of 51–53 kD and 48–50 kD, respectively. Note- the size marker appears to have run a bit slowly on the top gel, as the purified SHBG is slightly smaller than prior reports of 50–52 kD.

We addressed the question of the existence of SHBG isoforms in HepG2, LNCaP, and MCF-7 cells in initial Western blot studies. Immunoblot analysis of solubilized cellular proteins separated by SDS-PAGE, was performed using a polyclonal rabbit anti-human SHBG antibody (WAK-S1012-53, WAK-Chemie, Germany). Western blot analysis (Figure 8) shows that all three cell lines express an immunoreactive protein of the same molecular weight as purified SHBG. A lower molecular weight species of 33–35 kD was also detected (it could be greater because the molecular weight marker appears to have run slower than expected). To our knowledge, this is the first demonstration that such a lower molecular weight SHBG species exists in these cell lines. Since immunoreactive SHBG was not detected in the fetal bovine serum used in the prepared media (data not shown), we believe that this lower molecular weight species is either an isoform derived from one of the alternatively spliced SHBG transcripts, or a proteolytic fragment of SHBG itself. Assigning one of the transcripts described above to this species is difficult without further study, since SHBG has multiple glycoslyation sites that could increase its observed size by up to 6 kilodaltons, and a 29 amino acid long signal peptide at its amino terminus that may or may not be retained. Potential candidates include the 1L transcript lacking exons 6 and 7 which encodes an unmodified protein of 28.02 kilodaltons. If it exists, the exon 1L transcript lacking exon 4 encodes an unmodified protein of 37.87 kilodaltons; without the signal peptide it would have a molecular weight of 34.55 kilodaltons. Further studies should identify the origin of this novel smaller species. Compared to HepG2 and LNCaP, the appearance of multiple larger species in MCF-7 is likely due to differential glycosylation of SHBG itself [33,46].

## Discussion

**Figure 9:**
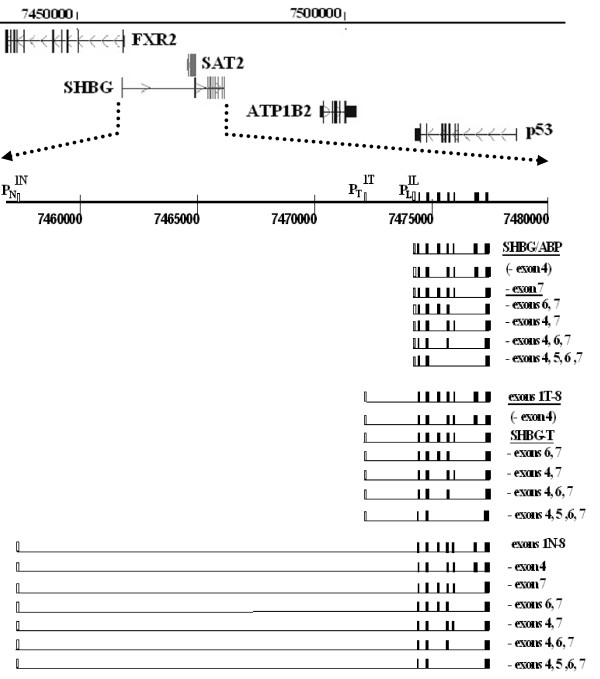
**The human SHBG gene locus on chromosome 17p13.1 and its transcriptional expression**. Top: Exon structures of the SHBG gene and its neighboring genes are given as vertical lines. Transcriptional polarity is denoted by arrows. The polarity (designated by arrows) of the SHBG gene is opposite that of the SAT2 and FXR2 genes. SHBG upstream exon 1T abuts the SAT2 gene, and exon 1N is located within intron 1 of the FXR2 gene. Note the close proximity of the p53 gene. Bottom: An expanded structure of the human SHBG gene, showing the three SHBG gene promoters, P_L_, P_T_, and P_N_, the three first exons, 1L, 1T, and 1N, and downstream exons 2–8. Numbers refer to relative positions along chromosome 17. Below is a representation of the structures of the 19 known human SHBG gene transcripts (boxes represent exons). Transcript structures are given on the right. Those representing the two major transcripts (SHBG/ABP and SHBG-T) and two minor transcripts (1L-8 lacking exon 7, and 1T-8) from prior reports are underlined. Alternative splicing of exon 4 from 1L-8 and 1T-8 transcripts was not specifically detected; these putative transcripts are presented in parentheses.

It is evident from these results that the current view of human SHBG gene structure, transcription, and, perhaps, protein expression requires modification. In addition to the two previously described human SHBG gene promoters, P_L_, and P_T_, we provide evidence for a novel, third upstream promoter, P_N_. Combined, these three promoters are responsible for generating at least 19 unique transcripts (Figure 9). To our knowledge, 15 of these 19 SHBG gene transcripts are novel, with the smaller sized SHBG mRNAs resulting from alternative splicing patterns that target exons 4, 5, 6, and/or 7.

### Alternative splicing of human SHBG transcripts

Certain nonannotated data in earlier studies on human SHBG gene expression are consistent with the existence of some of the novel, alternatively spliced human SHBG transcripts in this communication. Using an identical exon 5–8 RT-PCR assay, an uncharacterized small RT-PCR fragment was generated from ZR-75-1 and MDA-MB-231 cells (Fig. 1 in [17]), whose molecular size is consistent with the 176-bp fragment lacking exons 6 and 7 in our exon 5–8 RT-PCR assay (Figure 2A). This fragment is also present in our MCF-7 assay but was not apparent in theirs. Forges *et al*. (Figs. 2A and 2B in [47]) showed that human testis expresses the four earlier described SHBG transcripts; their data also include minor RT-PCR fragments that correspond to novel transcripts characterized in the present study. Selva *et al*. appear to generate at least one faint intermediate band between the contiguous exon 1T-8 and the exon 7 lacking RT-PCR fragments in human testis and sperm in their exon 1T-8 assay (Fig. 2B in [26]). More recently, Ng *et al*. used an exon 3–7 RT-PCR assay to demonstrate splicing of exon 4 alone [40]. In view of this new profile of human SHBG transcription and alternative splicing, conclusions from prior studies on SHBG expression based upon short-range RT-PCR assays may require reevaluation.

### Expression of the SHBG/ABP transcript

The notion that SHBG protein itself is expressed in certain hormonally responsive tissues is supported by the transcriptional profiles presented in this study, and our Western blot results. Human prostate [19] and breast [16], and specific regions of the brain [39] have previously been shown to display immunoreactive SHBG protein. Our 1L-8 RT-PCR assay showed that each of these tissues expresses the SHBG/ABP transcript. Compared to liver, the relative low expression of the transcript encoding SHBG in breast and prostate is consistent with a local effect/need for SHBG in acting on R_SHBG_. Extending this speculation, fibulin-1D and fibulin-2 have been shown to sequester plasma SHBG in the uterine stromal cells of transgenic mice, with a preference for steroid-bound SHBG [48]. Such an interaction could be envisioned as regulating local R_SHBG _signaling and/or sex steroid delivery in the human prostate, where SHBG expression predominates in luminal epithelial cells and R_SHBG _activity is highest in stromal cells [49].

It has been recently reported that SHBG acts to actively transport steroids into cells by binding of the endocytic receptor, megalin [50], though this finding has been disputed [51]. Should megalin serve such a function in the breast and prostate, then locally expressed SHBG could be a major player in steroid uptake in these tissues. Intracellular SHBG might also bind specific sex steroids, thereby influencing AR or ER activation. Therefore, locally expressed SHBG could play a role in a number of different biologic functions related to sex steroids.

### Biologic significance of alternatively spliced SHBG gene transcripts

We suspect that only specific transcripts derived from P_L _are likely candidates to encode stably translated SHBG isoforms. The exon 8 reading frame is retained in P_L _derived transcripts that lack exon 4 alone, exons 6 and 7, and exons 4, 6, and 7. The remaining P_L _derived transcripts, which present frameshifts within exon 8, appear less likely to be stably translated; an E326 SNP variant of SHBG, possessing a premature termination codon, is rapidly degraded [52] suggesting that motifs encoded within exon 8 confer protein stability [35]. We do not rule out the possibility that the exon 1L-8 transcript lacking exon 4 exists and went undetected due to aberrantly comigrating sequences, considering that we detected an exon 1N-8 transcript that lacks exon 4 from a mixture of comigrating RT-PCR fragments. Supporting this contention is a prior report that showed HepG2 transcripts to undergo splicing of exon 4 alone [40]. Based on its size alone, the 1L-8 transcript lacking exon 4 could encode the smaller, immunoreactive species recently described in human sperm [34].

Based upon their sequences, conventional translational initiation events can not explain how human P_N_- or P_T_- derived transcripts are translated into stable SHBG isoforms. The first potential AUG start codon within exon 1N heads a short, four amino acid open reading frame. The subsequent AUG codon lies at the beginning of a 32 amino acid open reading frame; a termination codon precludes its merge into the exon 2 SHBG/ABP reading frame. Exon 1T is devoid of an AUG start codon, the first potential AUG start codon within exon 2 of P_T_- derived transcripts occurs at the beginning of a short open reading frame consisting of only 10 amino acids. For such transcripts, it has been suggested that translation may initiate within exon 2 at a downstream AUG codon that shares the SHBG/ABP open reading frame, or that they may utilize alternative, rare start codons within exon 1T [34]. We note that, beginning with the CUG at position +13, there are eight possible alternative CUG, UUG, GUG, or AUA start codons [53] within the long exon 1T open reading frame that merges with the SHBG/ABP reading frame in exon 2. More work is required to determine the biologic fates of transcripts originating from P_T _and P_N_. Functionally, as has been seen with other genes [54], it is feasible that nontranslated transcripts originating from P_T _and P_N _could be regulatory in nature, affecting the expression of those that do encode SHBG and stable SHBG isoforms.

The 1L-8 transcripts lacking exons 6 and 7 or exon 4 alone could encode the smaller SHBG species we detected in HepG2, LNCaP and MCF-7 cells. A similar sized species was not identified in affinity purified SHBG from human plasma, hence, it might not bind steroids, and/or it may remain intracellular. The existence of an intracellular SHBG species that does not bind steroid, but retains the R_SHBG _membrane-binding motif within exon 3 [31] would be quite exciting, as it could potentially affect R_SHBG _signaling, as well as other SHBG mediated properties.

In addition to our finding of a smaller SHBG species in cell lines, human hypothalamus and cerebrospinal fluid have been shown to contain an immunoreactive SHBG species which is 4.3-kD smaller than that of serum SHBG [38]. Lower molecular weight species have been isolated from human male genital tissue and myocardium [55,56]. Taken together, these results suggest that a deeper investigation of alternatively spliced transcripts originating from P_L _may explain these findings.

### Human SHBG gene structure

The existence of human SHBG gene transcripts originating from P_T _and P_N _fits a larger picture in which the mammalian SHBG gene is in a state of evolutionary flux, 'hijacking' promoter elements from adjacent upstream genes on chromosome 17p13.1 that are transcribed in the opposite orientation (Figure 9). P_T_, which is located 1.4 kb upstream of P_L_, shares regulatory elements with the adjacent polyamine N-acetyltransferase (SAT2, NM_133491) gene. Based on chromosomal mapping of SHBG exon 1N, P_N _is located within intron 1 of the FXR2 gene. Such overlapping transcriptional units and bi-directional promoter arrangements have been described for a number of genes; computational studies of gene organization predict them to occur frequently within the human genome [57,58]. The rat possesses homologous exon 1N sequences (Figure 5A), but these have not been reported in mature rat SHBG transcripts. While prior work suggested that certain orthologous sequences might be incorporated into mature human transcripts, using RACE assays, we did not specifically detect the existence of a human homologue of rat P_A _[41,42]. Furthermore, the putative human exon 1A region contains no potential start codons (Figure 5B), thus, transcripts originating from a human P_A _would run into the same functional and translational questions as do transcripts derived from P_T_.

### Relative abundance of human SHBG transcripts in tissues and cell lines

Transcripts derived from P_L_, P_T_, and P_N _are expressed differentially in normal prostate, breast, liver, and testis. The relative expression of the SHBG/ABP transcript is consistent with our hypothesis regarding local generation of SHBG vs. the utilization of plasma SHBG- that immediate, rapid effects of SHBG, including those mediated by the R_SHBG _signaling pathway, are likely to be regulated through local expression of SHBG within responsive tissues; the concentration and/or availability of plasma SHBG is not responsive to SHBG needs in particular sites. Each of the cell lines we examined had a reduced abundance of total 1L transcripts as compared to their corresponding normal tissues, and more specifically this was the case for the SHBG/ABP transcript. Furthermore, the cancer cell lines examined in these studies had a greater overall abundance of alternatively spliced P_L _derived transcripts than did their corresponding normal tissues. If SHBG gene expression is indeed modulated in prostate and breast cancer, this could alter their androgen and estrogen responsiveness.

## Conclusion

In conclusion, our findings provide a broad and detailed picture of human SHBG transcript structure and expression. At the same time, they also raise many new and important questions, including the viability of novel alternatively spliced transcripts, their functions in hormonally responsive tissues, including the breast and prostate, and the role that aberrant SHBG gene expression may play in cancer.

## Methods

### Cell lines

The LNCaP FGC, MCF-7 and HepG2 cell lines were purchased from ATCC (Rockville, MD). LNCaP cells were maintained in RPMI 1640 (10-0410CV, Mediatech, Herndon, VA), supplemented with 10% fetal bovine serum (100–106, Gemini Bioproducts, West Sacramento, CA), 1 mM sodium pyruvate (Mediatech), 0.75% sodium bicarbonate (Mediatech), and 100 units/ml Penicillin-100 μg/ml Streptomycin (GIBCO, Grand Island, NY). The clonal MCF-7-Tr cell line, which constitutively expresses high levels of a transcript containing the full length human SHBG cDNA coding region, was generated by stably transfecting MCF-7 cells with the plasmid, pCMV-FLAG-SHBG. This plasmid contains the SHBG cDNA coding region (NM_001040) cloned downstream of a CMV promoter, and in frame with three iterations of an amino terminal FLAG tag. MCF-7 and MCF-7-Tr cells were maintained in DMEM (10-017-CV, Mediatech) supplemented with 10% fetal bovine serum, 1 mM sodium pyruvate, 0.75% sodium bicarbonate, 10 μM nonessential amino acids (Mediatech) and 100 units/ml Penicillin-100 μg/ml Streptomycin. HepG2 cells were grown in DMEM (10-010-CV, Mediatech) supplemented with 10% fetal bovine serum, 1 mM sodium pyruvate, 0.1 mM nonessential amino acids, 0.75% sodium bicarbonate and 100 units/ml Penicillin-100 μg/ml Streptomycin. Cells were refed every 2–3 days and split 1:3 to 1:5 upon attaining ≥80% confluence.

### RNAs

Total cellular RNAs were isolated from exponentially growing LNCaP, MCF-7, MCF-7-Tr, and HepG2 cells using TRIzol reagent (Invitrogen, Carlsbad, CA), according to the manufacturer's protocol. Total cellular RNAs were purchased from Ambion, Inc. (FirstChoice Total RNA; Applied Biosystems, Foster City, CA), these had been isolated from pooled normal testes (AM7972), liver (AM7960), prostate (AM7988), breast (AM6952), or brain (AM7962) tissues and DNase treated.

### Reverse Transcription-PCR (RT-PCR)

cDNA templates were generated using the cDNA cycle kit (Invitrogen) or Superscript III kit (Invitrogen), according to the manufacturer's protocol. One-two μg of RNA template were used in a total volume of 50 μl, containing 2.5 units of Platinum Taq polymerase (Invitrogen), 1× Platinum Taq buffer, 200 nM of each PCR primer, and 1–3 μl of cDNA. The primers used for PCR (Gene Link, Hawthorne, NY and MWG, Highpoint, NC) are presented [see Additional file 1]. PCR amplifications were performed using a denaturation step, 94°C for 10 minutes; followed by 35 cycles of denaturation at 94°C for 30 seconds, annealing (exon 5–8 assay: 54°C; exon 2–8 assay and exon 1N-8 assay: 56°C; exon 1L-8 assay and 1T-8 assay: 57°C) for 1 minute, and extension at 72°C for 2 minutes, followed by a final incubation at 72°C for 10 minutes. PCR products were analyzed by gel electrophoresis as before [19].

### Rapid Amplification of cDNA Ends (RACE)

RNA Ligase Mediated (RLM)-RACE was performed using the First Choice RLM-RACE kit (Ambion), according to the manufacturer's protocol. This kit is designed to amplify cDNA only from full-length, capped mRNA. Ten μg of total RNA from LNCaP, MCF-7, HepG2, and normal testis were used separately as starting material in standard reactions. The supplied 5' RACE adapter used for ligation to enzymatically decapped full length RNAs was: 5'-GCUGAUGGCGAUGAAUGAACACUGCGUUUGCUGGCUUUGAUGAAA-3'

Outer 5'RLM-RACE PCR was performed using the supplied 5'RACE outer primer and the SHBG exon 3 based gene specific outer primer, SHBG Reverse RACE Exon 3 [see Additional file 1]. Inner 5' RLM-RACE PCR was performed using the supplied 5'RACE inner primer and the SHBG exon 2 based gene specific inner primer, SHBG Reverse RACE Exon 2 [see Additional file 1]. RLM-RACE products were analyzed as above.

### DNA Sequencing

RT-PCR and RACE bands were stabbed with a sterile pipette tip, and transferred into 50 μl of 1× Platinum Taq buffer (Invitrogen); DNA fragments were eluted by 3 rounds of freeze-thawing. Five μl of each eluate were reamplified using original PCR conditions. Ten μl aliquots of each reamplified sample were analyzed by gel electrophoresis, and appropriate sized bands were cut out and eluted using the QIA quick gel extraction kit (Qiagen, Valencia, CA). Sequencing was performed at the Columbia University DNA Facility or at Genewiz Labs (Plainfield, NJ); both DNA strands were sequenced using the original PCR primers, and if necessary, nested primers.

### Quantitative RT-PCR (qPCR)

Total cellular RNAs were isolated from triplicate plates of exponentially growing HepG2, MCF-7 and LNCaP cells, treated with RNase-free DNase (Qiagen), and aliquots were analyzed by qPCR [59]. Duplicate aliquots of liver, testis, prostate, and breast RNAs were similarly tested. RNA aliquots were reverse transcribed to yield first-strand cDNAs using the Applied Biosystems Reverse Transcription Reagents protocol (Applied Biosystems) and products were diluted 1:10 in distilled H_2_O. Taqman assay PCR reactions (Perkin-Elmer-Applied Biosystems) were performed in triplicate in 96-well optical plates that included appropriate negative control reactions and wells, and run in an ABI PRISM 7700 Sequence Detection System machine. Assay-on-Demand Gene Expression Products (Applied Biosystems) were used; for individual reactions, 2.5 μl of each sample were combined with 12.5 μl of 2× Taqman Universal Master Mix, 1.25 μl of Target Assay Mix and 8.75 μl H_2_O. Predesigned Taqman assays for SHBG exons 2–3, 1L-2, and 1T-2 (Hs01050181_g1; Hs01050180_g1; and Hs01050186_m1) were purchased from Applied Biosystems. Assay by design primers (Applied Biosystems) SHBG exon 1N-1NF (forward primer), SHBG exon 1N-1NR (reverse primer), and SHBG exon 1N-1NM2 (probe) were used [see Additional file 1].

Data were extracted and amplification plots generated with ABI SDS software. Threshold cycle (Ct) scores, which represent the cycle number at which fluorescence signal crosses an arbitrary (user-defined) threshold, were averaged for subsequent calculations of relative expression values. For each of the four individual SHBG qPCR assays: 1) exon 2–3; 2) exon 1L-2; 3) exon 1T-2; and 4) exon 1N-2, and for each individual cell line and tissue, mean SHBG Ct scores were normalized against mean Ct scores for the endogenous control gene, glyceraldehyde-3-phosphate dehydrogenase (GAPDH), and standard deviations were calculated. Expression ratios for each assay were determined relative to MCF-7 expression by the following calculation: Relative Expression = 2^-ΔCt ^where ΔCt = normalized sample Ct – normalized MCF-7 Ct.

### Western Blot Analysis

HepG2, LNCaP, and MCF-7 cells were grown in 100 cm^2 ^dishes until they were approximately 80% confluent. After rinsing cells twice with cold PBS, two mls of protein extraction buffer (50 nM Tris Ph 8.0, 0.15 M NaCl, 0.5% NP40, 1× Halt Protease Inhibitor cocktail (#78430, Thermo Scientific)) were added to each plate, and left o/n on a shaker at 4°C. Cellular suspensions were collected and microfuged at 11,000 × g for 15 minutes at 4°C. Supernatants containing solubilized protein were transferred to sterile tubes, and protein concentrations were determined by Bradford assay (Biorad).

Equal amounts of protein (20 ug) from each cell line were loaded onto precast 10% LongLife gels (NH21-010, Gradipore-VWR), along with 10 pg of purified SHBG protein isolated from human plasma. Gel electrophoresis was performed at 75 volts in Tris-HEPES-SDS running buffer. Protein transfer onto PVDF membranes was performed at 15 volts for 30 minutes in an SD Transblot Cell (Biorad), using 25 mM Tris-base (pH 7.4), 192 mM Glycine, in 15% methanol as transfer buffer.

PVDF membranes were blocked by incubating overnight at 4°C in 20 mls of SuperBlock blocking buffer (Pierce Biotechnology, Rockford, IL) in Tris-buffered saline (pH 7.4) containing 0.5% Tween-20 (TBST) with gentle shaking. Membranes were then washed three times for five minutes each with 20 mls of SuperBlock Blocking Buffer/TBST with gentle shaking. Next, membranes were covered with 10 mls of SuperBlock Blocking Buffer/TBST containing a 1/1000 dilution of either a rabbit anti-hSHBG antibody (WAK-S1012-53, WAK-Chemie, Germany) or a rabbit anti-actin antibody (A2066, Sigma). Primary antibody incubation was for one hr at room temperature with gentle shaking, after which membranes were washed as above and covered with 10 mls of SuperBlock Blocking Buffer/TBST containing a 1/2000 dilution of Goat Anti-Rabbit IgG(H+L) conjugated to horseradish peroxidase (Cat. No. 31460, Pierce Biotechnology). Incubation with the secondary antibody was for one hr at room temperature with gentle shaking, after which membranes were washed as above, equilibrated in PBS, processed with Western Blotting Luminol Reagent (Cat. No. SC-2048, Santa Cruz Biotechnology, Santa Cruz, CA), and exposed to x-ray film.

## Abbreviations

The abbreviations used are: SHBG: sex hormone-binding globulin; R_SHBG_: SHBG receptor; ABP: androgen binding protein; P_L_: downstream promoter; P_T_: intermediate promoter; P_N_: novel upstream promoter; SHBG-T: major testis transcript (exon 1T-8, lacking exon 7); MCF-7-Tr: an MCF-7 clone stably transfected with a constitutively expressed SHBG cDNA construct.

## Competing interests

The authors declare that they have no competing interests.

## Authors' contributions

AMN performed RT-PCR experiments and purified fragments for DNA sequencing. DJH, WR, and NAR participated in the design of the study. ZX performed qPCR experiments. SMK conceived of the study, and participated in its design and coordination. All authors helped to draft the manuscript, and all authors read and approved the final manuscript.

## Supplementary Material

Additional file 1 **PCR primer sequences.** Click here for file
