# Supplementary material for: Human sex hormone-binding globulin gene expression- multiple promoters and complex alternative splicing
Source: BMC Mol Biol. 2009 May 5;10:37. doi: 10.1186/1471-2199-10-37 (PMC2694190; doi:10.1186/1471-2199-10-37)
Supplement: Additional file 1 — PCR primer sequences. [file 1471-2199-10-37-S1.doc]

RT-PCR primers:

Forward Primer: exon 5: 5’-ACTCAGGCAGAATCCAATCTC-3’

Reverse Primer: exon 8: 5’-CTTTAATGGGAAGCGTCAGT-3’

Forward Primer: exon 2: 5’-AGCAATGGCCCAGGACAA-3’

Forward Primer Exon 1L: 5’-TGGGCCCTGAGACCTGTTCT-3’

Forward Primer Exon 1T: 5’-GCAGTGATAACCTGCTTTAGCC-3’

Forward Primer Exon 1N: 5’-AATTCTCCATGTGCTTGGATCGT-3’

Reverse Primer Exon 1N: 5’- TCTTCCCCACGATCCAAG-3’

β-actin Forward Primer: 5’-ATCTGGCACCACACCTTCTACAATGAGCTGCG-3’

β-actin Reverse Primer: 5’-CGTCATACTCCTGCTTGCTGATGCACATCTGC-3’

5’ RACE Outer Primer: 5’-GCTGATGGCGATGAATGAACACTG-3’

SHBG Reverse RACE Exon 3: 5’-TGGTGCCATCTCCCATCATCCA-3’

5’ RACE Inner Primer: 5’-CGCGGATCCGAACACTGCGTTTGCTGGCTTTGATG-3’

SHBG Reverse RACE Exon 2: 5’-TCTTGTCCTGGGCCATTGCTGA-3’

Taqman qPCR primer sets:

SHBG exons 2-3: ABI cat. Hs01050181_g1

SHBG exons 1L-2 : ABI cat. Hs01050180_g1

SHBG exons 1T-2: ABI cat. Hs01050186_m1

Taqman Assay by Design Taqman qPCR primer set:

SHBG exon 1N-1NF: 5’-AATTCTCCATGTGCTTGGATCGT-3’

SHBG exon 1N-1NR: 5’-TTGTCCTGGGCCATTGCT-3’

Probe: SHBG exon 1N-1NM2: FAM-labeled-5’-TCGTGGGCACTCTCTT-3’
